# Outcomes for older people with screening-detected versus existing chronic kidney disease: a cohort study with data linkage

**DOI:** 10.3399/BJGPO.2024.0123

**Published:** 2025-02-26

**Authors:** Anna K Forbes, José M Ordóñez-Mena, Winnie Mei, Clare J Taylor, Nicholas Jones, Jennifer A Hirst, FD Richard Hobbs

**Affiliations:** 1 Nuffield Department of Primary Care Health Sciences, University of Oxford, Oxford, UK; 2 National Institute for Health and Care Research Applied Research Collaboration Oxford and Thames Valley, Oxford Health NHS Foundation Trust, Oxford, UK; 3 Institute of Applied Health Research, University of Birmingham, Birmingham, UK

**Keywords:** screening, renal medicine, aged, renal insufficiency, chronic, primary health care

## Abstract

**Background:**

Chronic kidney disease (CKD) is a common health problem associated with increased risk of cardiovascular disease (CVD), end-stage kidney disease (ESKD), and premature death. It is estimated that one-third of people aged ≥70 years have CKD globally, many of whom are undiagnosed, but little is known about the value of screening.

**Aim:**

To compare the risk of adverse health outcomes between people with an existing diagnosis of CKD and those identified through screening, and identify factors associated with mortality in CKD.

**Design & setting:**

Prospective cohort study of 892 primary care patients aged ≥60 years with CKD (existing and screening detected) in Oxfordshire, with data linkage to civil death registry and secondary care.

**Method:**

Hazard ratios (HRs) and 95% confidence intervals (CIs) were estimated using Cox proportional hazard models to compare the risk of all-cause mortality, hospitalisation, CVD, ESKD separately, and as a composite between CKD groups, as well as to identify factors associated with mortality.

**Results:**

After a median follow-up of 3–5 years, 49 people died, 512 were hospitalised, 78 had an incident CVD event, and none had an ESKD event. There was no difference in the composite outcome between those with existing CKD and those identified through screening (HR 0.94, 95% CI = 0.67 to 1.33). Older age (HR 1.10, 95% CI = 1.06 to 1.15), male sex (HR 2.31, 95% CI = 1.26 to 4.24), and heart failure (HR 5.18, 95% CI = 2.45 to 10.97) were associated with increased risk of death.

**Conclusion:**

Screening older people for CKD may be of value, as their risk of short-term mortality, hospitalisation, and CVD is comparable with people routinely diagnosed. Larger studies with longer follow-up in more diverse and representative populations of older adults are needed to corroborate these findings.

## How this fits in

Chronic kidney disease (CKD) is a common health problem in older adults, estimated to affect up to 35% of people aged ≥70 years. CKD is associated with a significantly increased risk of cardiovascular disease (CVD) and premature death. Many people are living with undiagnosed CKD, but little is known about the potential benefit of screening older people for the condition. Our findings show that the risk of short-term mortality, hospitalisation, and CVD is comparable in people diagnosed through screening with those diagnosed routinely in primary care. This suggests that screening older people for CKD may be of value to increase detection and enable disease-modifying treatment to be initiated at an earlier stage. Larger studies with longer follow-up in more diverse and representative populations of older adults are needed to corroborate these findings.

## Introduction

CKD is a common and increasing health problem, with an estimated prevalence of 13.9% in adults in England.^1^ This is largely attributed to the ageing population and rising prevalence of type 2 diabetes and hypertension.^2,3^ CKD is a heterogenous group of disorders characterised by an estimated glomerular filtration rate (eGFR) <60 ml/min/1.73 m^2^ and/or other measures of kidney damage, including a urine albumin-to-creatinine ratio (ACR) ≥3 mg/mmol, present for at least 3 months.^4^ CKD is a powerful and independent risk factor for CVD and premature death.^5,6^ While most people with CKD will not develop end-stage kidney disease (ESKD) in their lifetime, the burden of CVD is substantial, even in early stages of CKD. Early detection of CKD is recommended to address cardiovascular risk, slow CKD progression, and reduce those developing ESKD.^7^ This can be achieved through blood pressure control, renin-angiotensin system (RAS) inhibitors, and sodium-glucose co-transporter 2 (SGLT2) inhibitors, which have been shown to prevent adverse cardiovascular and kidney outcomes in people with CKD.^8,9^

CKD is often asymptomatic, particularly in the early stages, and consequently many people are living with the condition undiagnosed.^10,11^ Screening for CKD can be useful in identifying unrecognised cases, but full population screening is low yield and may not be cost-effective.^12,13^ Screening programmes in high-risk groups, including those with diabetes and hypertension, are well established.^4,14^ It is estimated that up to 35% of people aged ≥70 years are living with CKD in part owing to age-related decline in kidney function.^3^ However, despite this high prevalence, targeted CKD screening of older adults is not routinely implemented in clinical practice, and its potential value has not been well explored.

In 2013, the Oxford Renal (OxRen) cohort study was established to screen an older population of people in primary care for CKD.^15^ This study showed that nearly half of people with CKD (44%) had not previously been diagnosed.^10^ The present study aimed to link OxRen data with clinical outcomes from NHS Digital to determine the value of screening for CKD in an older primary care population. The aim was to compare the risk of adverse health outcomes between people with an existing diagnosis of CKD and those with screening-detected CKD. The primary objective was to compare the risk of all-cause mortality, hospitalisation, CVD, and ESKD between these CKD groups. The secondary objective was to identify factors associated with adverse health outcomes in patients with CKD.

## Method

### Study design and study population

The OxRen study was the largest national CKD observational study, recruiting 3207 individuals aged ≥60 years from primary care practices in Oxfordshire, England, from November 2013–July 2017.^15^ Individuals entered the OxRen CKD cohort either based on an existing diagnosis of CKD in their primary care record or through screening. Screening-detected CKD was defined by an eGFR <60 ml/min/1.73 m^2^ and/or a urine ACR ≥3 mg/mmol, which persisted on repeat measurement 3 months later. Screening also identified a group of individuals with transiently impaired kidney function whose eGFR and/or urine ACR returned to normal at the second screening visit. Detailed methodology of the OxRen study is described elsewhere.^10,15^

The present study, known as the New onset Kidney Impairment (NewKI) study, recruited participants from the OxRen CKD cohort. Individuals were invited to take part in a baseline assessment and to provide written informed consent for their NHS number and date of birth to be linked to the Office for National Statistics civil death registry and Hospital Episode Statistics (HES) Admitted Patient Care data. These data were provided by NHS Digital and enabled us to follow participants over time to conduct a population-based prospective cohort study.

### Outcome measures

The primary outcome measure was a composite of all-cause mortality, hospitalisation for any cause, CVD, or ESKD. Secondary outcome measures included each individual component of the composite primary outcome measure. Additional outcome measures were factors associated with mortality in people with CKD.

The primary outcome measures were reported according to CKD subgroup, which were defined as follows: 1) people with an existing diagnosis of CKD (existing CKD); 2) people with newly diagnosed CKD after screening (screening-detected CKD); and 3) people with transiently reduced kidney function identified through screening (transiently impaired kidney function).

### Outcome ascertainment

Information concerning vital status and cause of death was ascertained until 2 July 2021. Owing to a lag in HES data, CVD events were ascertained until 21 March 2020 and ESKD events were ascertained until 30 November 2019. *International Classification of Diseases, 10th revision* (ICD-10) codes were used to define CVD and ESKD events (code lists are shown in Supplementary Appendix S1 and S2, respectively). All CVD and ESKD events recorded before study entry were considered as prevalent and excluded from analysis of the incidence.

### Factors associated with mortality

We investigated the association between various characteristics and mortality in people with CKD. This included the following sociodemographic and lifestyle variables: age; sex; ethnicity (White versus non-White); body mass index; waist-to-hip ratio; smoking status (never, former, or current); alcohol intake (g per week); and level of education. Level of education was coded as follows: primary only (if the participants answered they had no qualifications); secondary (if the participants answered they had A-levels, GCSEs, or O-levels); and higher education (if the participants answered they had university or postgraduate studies). We also investigated blood pressure and comorbidities including hypertension, diabetes, ischaemic heart disease, heart failure, atrial fibrillation, peripheral vascular disease, other cardiovascular disease, renal disease, urinary tract infection, thyroid disease, anaemia, osteopenia, and osteoporosis.

### Statistical analysis

Sociodemographic, lifestyle, and comorbidity variables were tabulated and reported as median and interquartile range (IQR) for continuous variables, or frequency and percentage for categorical variables. These baseline characteristics were reported separately for each CKD subgroup.

Cox proportional hazards models were used to estimate hazard ratios (HRs) and 95% confidence intervals (CIs) for the association of CKD subgroups and other factors associated with all-cause mortality, hospitalisation for any cause, CVD, and ESKD. Kaplan–Meier (KM) curves and log-rank tests were used to compare survival between CKD subgroups, age groups, and male and female.

All analyses were conducted with R (version 4.2.0) using the ‘survival’ and ‘survminer’ packages.

### Power calculation

A previous UK observational study reported that 69% of people routinely diagnosed with new CKD had died by the end of study follow-up at 5.5 years.^16^ In the absence of UK data for people with CKD detected by screening, we used data from a French cohort of patients testing positive for CKD in two measurements 3 months apart. The mortality in this cohort was 38% after 3 years of follow-up.^17^ Using the sample size for the existing CKD and screening-detected CKD groups in our study, and a 5% level of statistical significance, we estimated our statistical power to be sufficient (>99%) to detect such difference in mortality between groups. We expected this difference to be much lower after adjustment for age and other determinants of mortality. However, even if the ratio between both groups was as low as 1.27, we would still have >80% power. Smaller differences in the risk of death may suggest screening is helpful at identifying people with similar risk of death as those already diagnosed routinely.

## Results

In total, 902 participants consented for linkage of their data to NHS Digital. Data for 892 participants were available for the analysis, of whom 257 (28.8%) had an existing diagnosis of CKD, 185 had screening-detected CKD (20.7%), and 450 (50.4%) had transiently reduced kidney function.

The characteristics of the study participants by CKD subgroup are shown in Table 1. There was a higher proportion of females in the existing CKD group (53.3%), while in the screening-detected CKD group there was a higher proportion of males (54.1%). The median eGFR was lower in those with an existing diagnosis of CKD, compared with those identified through screening (55.0 ml/min/1.73 m^2^ versus 65.0 ml/min/1.73 m^2^). Most patients did not have albuminuria, and median urine ACR was similar across CKD groups (Existing CKD 2.45 mg/mmol and screening-detected CKD 2.80 mg/mmol). People with an existing CKD diagnosis had a higher burden of hypertension, diabetes, and ischaemic heart disease, compared with those with screening-detected CKD.

**Table 1.** Baseline characteristics of people with CKD stratified by CKD category

Variable	Existing CKD, *n* = **257**		Screening-detected CKD, *n* = **185**		Transiently impaired kidney function, *n* = **450**	
**Sex, *n* (%)**						
Male	120	(46.7)	100	(54.1)	205	(45.6)
Female	137	(53.3)	85	(45.9)	245	(54.4)
**Age, years, median (IQR)**	75.0	(70.5–79.0)	73.7	(69.4–77.8)	72.9	(69.1–78.3)
**Smoking status, *n* (%)**						
Never	133	(51.8)	97	(52.4)	256	(56.9)
Former	116	(45.1)	77	(41.6)	180	(40.0)
Current	8	(3.1)	11	(5.9)	14	(3.1)
**Ethnicity, *n* (%)**						
White	253	(98.4)	181	(97.8)	444	(98.7)
Non-White	4	(1.6)	4	(2.2)	6	(1.3)
**Education, *n* (%)**						
Primary only	87	(33.9)	71	(38.4)	153	(34.0)
Secondary	97	(37.7)	68	(36.8)	170	(37.8)
Higher education	73	(28.4)	46	(24.9)	127	(28.2)
**Medical history of, *n* (%)**						
Hypertension	166	(64.6)	107	(57.8)	224	(49.8)
Diabetes	56	(21.8)	22	(11.9)	54	(12.0)
Ischaemic heart disease	57	(22.2)	27	(14.6)	69	(15.3)
Heart failure	10	(3.9)	9	(4.9)	15	(3.3)
Atrial fibrillation	41	(16.0)	15	(8.1)	47	(10.4)
Other cardiovascular disease	22	(8.6)	9	(4.9)	27	(6.0)
Peripheral vascular disease	9	(3.5)	8	(4.3)	20	(4.4)
Renal disease	216	(84.0)	43	(23.2)	64	(14.2)
UTI	108	(42.0)	75	(40.5)	205	(45.6)
Thyroid disease	32	(12.5)	21	(11.4)	54	(12.0)
Anaemia	30	(11.7)	16	(8.6)	46	(10.2)
Osteopenia	20	(7.8)	13	(7.0)	24	(5.3)
Osteoporosis	18	(7.0)	13	(7.0)	34	(7.6)
**Alcohol intake, g, median (IQR)**	2	(1–7)	4	(1–10)	3	(0–10)
**BMI, kg/m^2^, median (IQR)**	26.90	(24.69– 30.45)	27.22	(24.32–30.86)	26.85	(23.75–30.64)
**WHR, median (IQR)**	0.92	(0.84–0.97)	0.91	(0.85–0.98)	0.90	(0.84–0.97)
**Systolic blood pressure, mmHg, median (IQR)**	131	(120–144)	136	(124–149)	135	(123–146)
**Diastolic blood pressure, mmHg, median (IQR)**	78	(70–85)	80	(72–86)	80	(72–87)
**eGFR, ml/min/1.73 m^2^, median (IQR)**	55.0	(45.9– 63.9)	65.0	(57.0–77.7)	67.0	(59.0–80.0)
**Urine ACR, mg/mmol, median (IQR)**	2.45	(0.80–2.50)	2.80	(1.95–7.50)	2.45	(1.10–2.50)

ACR = albumin-to-creatinine ratio. BMI = body mass index. CKD = chronic kidney disease. eGFR = estimated glomerular filtration rate. IQR = interquartile range. UTI = urinary tract infection. WHR = waist-to-hip ratio.

### All-cause mortality

In the overall cohort, after a median 4.59 (IQR 3.69–5.34) years of follow-up, 49 (5.5%) study participants died. The probability of survival beyond 5 years was 0.94 (95% CI = 0.92 to 0.96). The leading cause of death was cancer, accounting for 44.9% of all deaths (*n* = 22), followed by CVD, which contributed to 26.5% of the deaths (*n* = 13) (data not shown).

**Figure 1.**
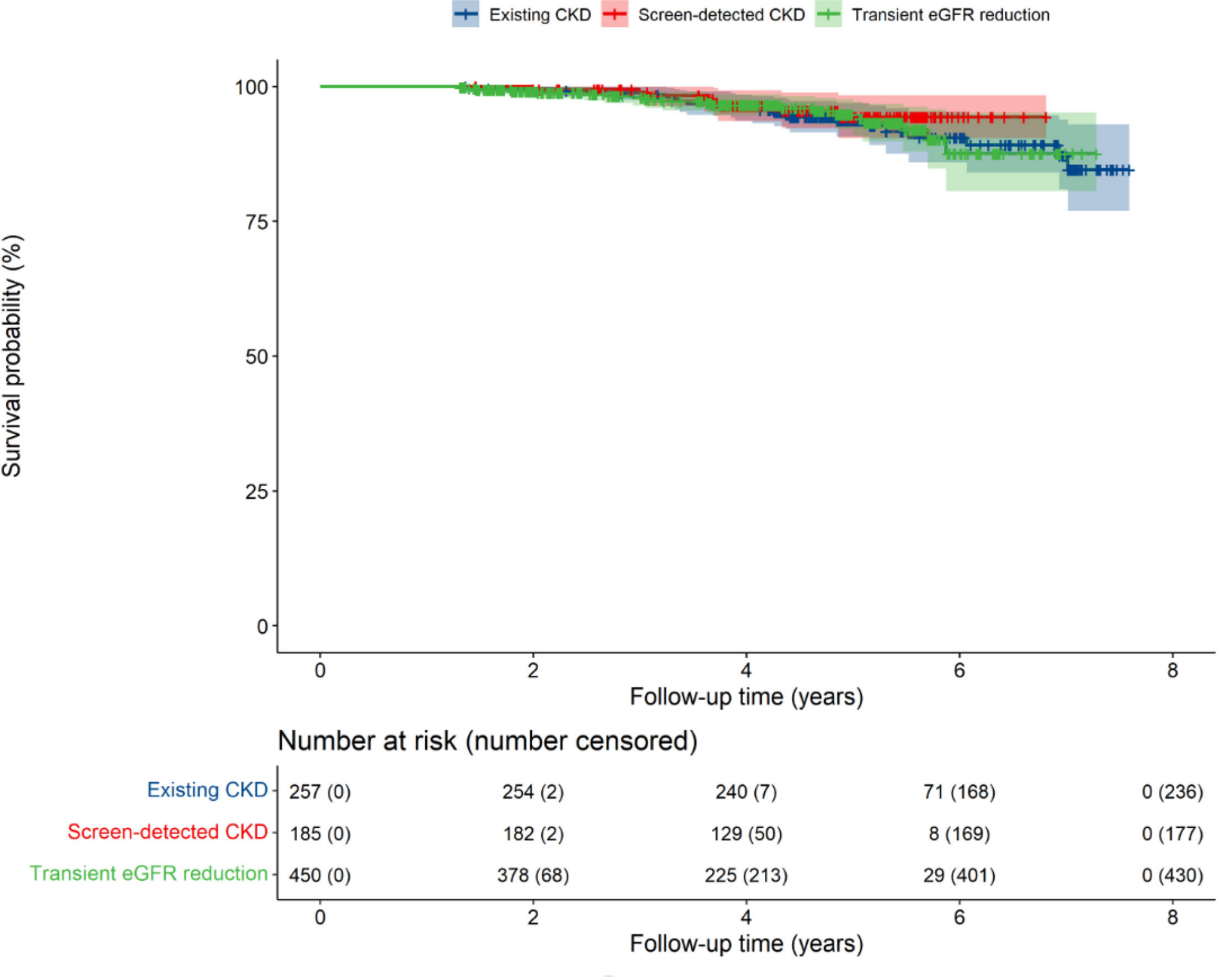
Kaplan–Meier survival probability over time stratified by CKD group. CKD = chronic kidney disease. eGFR = estimated glomerular filtration rate.

There was no difference in all-cause mortality between CKD subgroups before or after adjustment for confounders (Table 2). Figure 1 illustrates the KM survival probability over time stratified by CKD group.

**Table 2.** Association of CKD categories with all-cause mortality among participants in the NewKI study

Category	Median follow-up, years	PYs	*N*	*n*	MR	Crude model, HR (95% CI)		Age and sex adjusted, HR (95% CI)		Multivariable model, HR (95% CI)^a^	
Existing CKD	4.93	1350	257	21	15.56	1.00	(Reference)	1.00	(Reference)	1.00	(Reference)
Screening-detected CKD	4.57	838	185	8	9.55	0.74	(0.32 to 1.69)	0.81	(0.35 to 1.87)	0.41	(0.11 to 1.56)
Transiently impaired kidney function	4.00	1756	450	20	11.39	0.95	(0.51 to 1.78)	1.16	(0.61 to 2.19)	0.96	(0.35 to 2.65)

a Multivariable model was adjusted for age, sex, smoking status, and medical history of diabetes, hypertension, ischaemic heart disease, heart failure, other cardiovascular disease, estimated glomerular filtration rate, and urine albumin-to-creatinine ratio. CKD = chronic kidney disease. HR = hazard ratio. MR = mortality rate (that is, the quotient of number of deaths by the sum of follow-up time at risk). *N* = number of participants in the category. *n* = number of deaths in the category. NewKI = New onset Kidney Impairment. PYs = sum of follow-up time at risk in years for all participants in the category.

Supplementary Table S1 shows estimates of the factors associated with all-cause mortality in people with CKD, including those with an existing diagnosis of CKD and screening-detected CKD. Males with CKD had more than twice the risk of dying from any cause as females with CKD (HR 2.50, 95% CI = 1.36 to 4.60). The association of sex with all-cause mortality remained statistically significant even after adjusting for age (HR 2.31, 95% CI = 1.26 to 4.24). Age was also associated with all-cause mortality in people with CKD. Per each additional year of age, the risk of death increased by 11% (HR 1.11, 95% CI = 1.06 to 1.15). The association of age with all-cause mortality was not attenuated following adjustment for sex (HR 1.10, 95% CI = 1.06 to 1.15). Supplementary Figures S1 and S2 illustrate the KM survival probability over time stratified by sex and age.

A person with CKD and a medical history of heart failure had a large increase in the risk of all-cause mortality (HR 8.19, 95% CI = 3.94 to 16.99) compared with those with CKD but without heart failure. After adjustment for age and sex, the association persisted but was attenuated (HR 5.18, 95% CI = 2.45 to 10.97) (see Supplementary Table S1).

### Cardiovascular disease and end-stage kidney disease

Before enrolment in the study, 57 (6.4%) participants had already had a CVD event and were therefore excluded from the incident analysis. After a median follow-up of 3.06 years (IQR 1.63–3.88), 78 (9.3%) study participants had an incident CVD event (see Supplementary Table S2). There was no difference in CVD incidence between CKD subgroups before or after adjustment for confounders. No participants had an ESKD event during the study period. No further analysis was therefore conducted for this outcome.

### Hospitalisation

After a median follow-up of 1.62 (IQR 0.57–3.14) years, 512 (57.4%) of the study participants had a hospitalisation event. There was no difference in hospitalisation incidence between CKD subgroups before or after adjustment for confounders (see Supplementary Table S3).

### Composite outcome

After a median follow-up of 1.62 (IQR 0.57–3.14) years, 515 (57.7%) of the study participants had a composite outcome event. There was no difference in the incidence of the composite outcome between CKD subgroups before or after adjustment for confounders (fully adjusted HR 1.04, 95% CI = 0.76 to 1.42). The results from the crude, adjusted, and fully adjusted models are shown in Table 3.

**Table 3.** Association of CKD categories with the composite outcome among participants in the NewKI study

Association of CKD categories with the composite outcome among participants in the NewKI study.											
Category	Median follow-up (years)	PYs	N	n	IR	Crude model		Age & Sex adjusted		Multivariable model *	
Existing CKD	2.18	602	257	177	294	1.00	(Reference)	1.00	(Reference)	1.00	(Reference)
Screen-detected CKD	1.88	361	185	120	332.6	1.10	(0.87 to 1.38)	1.15	(0.91 to 1.46)	1.04	(0.76 to 1.42)
Transient eGFR reduction	1.24	750	450	218	290.8	0.94	(0.77 to 1.15)	1.01	(0.83 to 1.23)	0.99	(0.73 to 1.35)

a Multivariable model was adjusted for age, sex, smoking status, and medical history of diabetes, hypertension, ischaemic heart disease, heart failure, other cardiovascular disease, estimated glomerular filtration rate, and urine albumin-to-creatinine ratio. CKD = chronic kidney disease. IR = incidence rate, per 1000 person–years (that is, the quotient of number of composite outcome cases by the sum of follow-up time at risk). HR = hazard ratio. *N* = number of participants in the category. *n* = number of composite outcome cases in the category. PYs = sum of follow-up time at risk in years for all participants in the category.

## Discussion

### Summary

In this unique prospective cohort of older adults in English primary care, we found no difference in short-term mortality, hospitalisation owing to any cause, or CVD incidence between people with an established diagnosis of CKD and those identified through screening.

Older age (per 1 year fully adjusted HR 1.11, 95% CI = 1.06 to 1.15), male sex (fully adjusted HR 2.31, 95% CI = 1.26 to 4.24), and heart failure (fully adjusted HR 5.18, 95% CI = 2.45 to 10.97) were associated with increased risks of death in people with CKD.

### Strengths and limitations

To our knowledge, this is the first study to compare short-term health outcomes in older primary care populations who were screened for CKD. A significant strength of this study is that CKD was diagnosed using Kidney Disease Improving Global Outcomes (KDIGO) guidance.^18^ Our CKD screening process included an assessment of both eGFR and proteinuria, which was repeated after 3 months. Outcomes were assessed through data linkage with NHS Digital, enabling us to access data on deaths and hospitalisations, which are highly regarded in terms of their quality.

Although the magnitude of the observed difference in mortality between the CKD groups was as anticipated, the observed mortality rate in the cohort was lower than expected. This may have impacted our ability to detect a difference in mortality between those with existing CKD diagnosis and those with screening-detected CKD. Moreover, owing to a relatively short follow-up period and a cohort of patients with early stages of CKD, we did not identify any cases of ESKD.

Selection bias is a limitation of our study. The study population was comprised largely of people of White ethnicity, which has implications for the generalisability of our findings to the broader population of older adults both in the UK and other countries. Ethnicity is an established risk factor for mortality in people with CKD.^19^ Nearly all the participants in our study were of White ethnicity, and this prevented us from investigating associations of ethnicity with death and other outcomes. This may have also led to a lower overall mortality rate in the cohort.

Individuals were recruited into the present study based on the CKD groups they were originally assigned in the OxRen study; existing CKD, screening-detected CKD, and screening-detected transiently impaired kidney function. Kidney function may have changed over time and those with transiently impaired kidney function may have progressed to CKD. This may have resulted in misclassification bias, impacting on our ability to detect differences between the groups.

Confounding is a potential limitation of our analysis exploring factors associated with mortality in people with CKD. We adjusted for age and sex in the models, but it is possible that other variables, including cardiovascular comorbidities, prescribed medications, eGFR, and urine ACR, confounded the associations we observed.

### Comparison with existing literature

Previous screening programmes have identified large numbers of people with undiagnosed CKD,^20^ but population-based screening has not been shown to be cost-effective.^12,13^ Targeted screening of high-risk groups, including those with diabetes and hypertension, is recommended in many healthcare settings.^4,14^ Several systematic reviews have evaluated the effectiveness of targeted CKD screening in the community.^13,21^ These reviews identified significant variation in the assessment of CKD, with many screening programmes not following KDIGO guidance on diagnosing CKD, relying on either a single abnormal result or eGFR alone. This has limited the value of these programmes in drawing conclusions on the effectiveness of screening. Moreover, new interventions, such as SGLT2 inhibitors, have recently been recommended for use in people with CKD, warranting a re-evaluation of the potential benefits and cost-effectiveness of CKD screening.^22^

Older adults are not routinely screened for CKD and its potential value has not previously been extensively explored. A cost-utility analysis of a Canadian CKD screening programme, using assessment of eGFR, found that it was not cost-effective overall or in older adults.^12^ They explored the impact of screening on the risk of ESKD and mortality. The analysis was based on the premise that screening would be expected to identify patients with no previous diagnosis who could then receive an RAS inhibitor. In subgroups of people aged <65 and ≥65 years, the cost per quality-adjusted life year (QALY) gained associated with screening was $200 100 (Canadian dollars; approximately £116 675) and $93 700 (approximately £54 635), respectively. Conversely, in subgroups of people with and without diabetes, the cost per QALY gained was $22 600 (approximately £13 177) and $572 000 (approximately £333 525), respectively.^12^ However, this study used eGFR to diagnose CKD and potentially failed to identify individuals with proteinuria, who are at higher risk of adverse outcomes and more likely to benefit from intervention. The study was also published before the availability of SGLT2 inhibitors, and their potential impact was subsequently not considered in the analysis.

### Implications for research and practice

Our study suggests that screening older people in primary care for CKD may be of value, as the risk of mortality and other adverse health outcomes are comparable with those with an established diagnosis of CKD. Individuals with transiently impaired kidney function also had comparable outcomes, likely reflecting a group at higher risk of adverse outcomes than those with normal kidney function. Understanding what happens to the kidney function of those with transiently impaired kidney function over time would be valuable and the lack of follow-up data on the kidney function in this group is a limitation of the present study.

Older patients with CKD have several long-term conditions and this requires holistic care from the GP. There was no difference in cardiovascular events between subgroups and the main cause of death overall was cancer. The frequency of comorbidities in this age group may be the reason why there is no difference seen in outcomes. The group with heart failure were at particularly high risk and may be a group most likely to benefit from screening for CKD to optimise management.

The screening-detected CKD population we identified had relatively preserved eGFR and few had albuminuria. This could limit the potential of this group to benefit from interventions, such as RAS inhibitors and SGLT2 inhibitors, given that their risk of CVD and progressive CKD will be comparatively lower than other groups with high CKD prevalence, such as those with diabetes.

Our study population was small and not representative of the broader population of older adults in the UK. The characteristics of a larger and more diverse screening-detected older CKD population needs to be established, including the prevalence of proteinuric kidney disease and proportions of those with more advanced kidney disease. This would help to determine cardiovascular risk and likelihood to benefit from interventions such as RAS inhibitors and SGLT2 inhibitors in higher risk patients. Future studies therefore need larger, older, and more diverse study populations with longer follow-up, to determine whether CKD screening is cost-effective and improves clinical outcomes and quality of life for older adults.

In conclusion, our findings show that the risk of short-term mortality, hospitalisation, and CVD is comparable in older people with CKD diagnosed through screening with those diagnosed routinely in primary care. This suggests that screening older people for CKD may be of value to increase detection and enable disease-modifying treatment to be initiated at an earlier stage. Larger studies with longer follow-up in more diverse and representative populations of older adults are needed to corroborate these findings and determine whether CKD screening is effective in this group.

## Data

The datasets used and/or analysed during the current study are available from the corresponding author on reasonable request.
